# Four-Dimensional Image-Guided Adaptive Brachytherapy for Cervical Cancer: A Systematic Review and Meta-Regression Analysis

**DOI:** 10.3389/fonc.2022.870570

**Published:** 2022-07-04

**Authors:** Fei Li, Dan Shi, Mingwei Bu, Shuangchen Lu, Hongfu Zhao

**Affiliations:** ^1^ Department of Radiation Oncology, China-Japan Union Hospital of Jilin University, Changchun, China; ^2^ Department of Radiation Oncology, Guowen Medical Corporation Changchun Hospital, Changchun, China; ^3^ Department of Radiation Oncology, Second Affiliated Hospital of Jilin University, Changchun, China

**Keywords:** cervical cancer, image-guided adaptive brachytherapy, systematic review, dose-effect relationship, brachytherapy

## Abstract

**Purpose:**

The ICRU/GEC-ESTRO released the ICRU Report No. 89, which introduced the concept of four-dimensional brachytherapy and ushered in a new era of brachytherapy for cervical cancer. The purpose of this study was to evaluate the local control and late toxicity of four-dimensional brachytherapy in cervical cancer through a systematic review and to reveal the dose-response relationship between the volumetric dose paraments and the local control rate *via* a probit model.

**Material and Methods:**

We identified studies that reported the HR-CTV D90 and local control probabilities by searching the PubMed Database, the Web of Science Core Collection and the Cochrane Library Database through February 1st, 2022. Regression analyses were performed between the HR-CTV D90 and the local control probability using a probit model.

**Results:**

Nineteen studies enrolling 3,616 patients were included. The probit model showed a significant relationship between the HR-CTV D90 value and IR-CTV D90 Vs. the local control probability, P < 0.001 and P = 0.003, respectively. The D90 for HR-CTV and IR-CTV corresponding to a probability of 90% local control was 79.1 GyEQD2,10 (95% CI:69.8 – 83.7 GyEQD2,10) and 66.5 GyEQD2,10 (95% CI: 62.8 - 67.9 GyEQD2,10), respectively. The limits for the prescribed dose of 85 GyEQD2,10 for HR-CTV D90 theoretically warranted a 92.1% (95% CI: 90.2% - 95.3%) local control rate, and 87.2% (95% CI: 82.4% - 91.8%) local control probability was expected for 65 GyEQD2,10 to IR-CTV D90. The probit model showed no significant relationship between the D2cc to organs at risk and the probability of grade 3 and above gastrointestinal or genitourinary toxicity.

**Conclusions:**

Four-dimensional brachytherapy takes into account uncertain factors such as tumour regression, internal organ motion and organ filling, and provides a more accurate and more therapeutic ratio delivery through adaptive delineation and replanning, replacement of the applicator, and the addition of interstitial needles. The dose volume effect relationship of four-dimensional brachytherapy between the HR-CTV D90 and the local control rate provides an objective planning aim dose.

## Purpose

The use of brachytherapy in the treatment of cervical cancer has increased worldwide since its initial introduction over 110 years ago. Brachytherapy can provide a high dose to the tumor with excellent sparing to the organs at risk (OARs) due to the steep dose gradient. Before the development of external beam radiation therapy (EBRT), brachytherapy played an important role in the local treatment of cervical cancer. With the development of new EBRT technologies, such as intensity-modulated radiation therapy and stereotactic body radiotherapy, highly conformal doses can be provided. However, brachytherapy is still an irreplaceably important part of radiotherapy for cervical cancer, which improves the overall survival rate and disease-related survival rate (1). Concomitant chemoradiation followed by brachytherapy is the standard treatment for locally advanced cervical cancers. Compared with conventional brachytherapy, three-dimensional image-guided brachytherapy (3D-IGBT) improves the survival of patients with cervical cancer with similar or lower incidences of side effects (2–6).

Based on the wide application of 3D-IGBT for cervical cancer, the ICRU/GEC-ESTRO released ICRU Report No. 89, which focused on adaptive brachytherapy. It introduced the concept of four-dimensional (4D) image-guided adaptive brachytherapy (IGABT). 4D is three spatial dimensions and time. 4D-IGABT aims to improve the efficacy-to-toxicity ratio by exploiting the tumor-volume regression often seen in cervical cancer after the first phase of treatment. The introduction of the concept of 4D-IGABT is ushering in a new era of brachytherapy for cervical cancer (7).

Our previous systematic review and probit model analysis focused on intracavitary combined interstitial brachytherapy (8). Although the adaptive method was adopted to some extent in the enrollment study of reference 8, their focus was not 4D-IGABT. Therefore, most studies (27 of 33) had neither “adaptive” nor “IGABT” in the title, abstract, or keywords of the article. This study is a data update for reference (8) and focuses on the 4D-IGABT. The purpose of this study was to evaluate the local control of 4D-IGABT in cervical cancer through a systematic review. A regression analysis between the dose-volume histogram parameters and the local control rate was carried out *via* a probit model.

## Material and Methods

We performed a comprehensive literature search using the PubMed database, the Web of Science Core Collection, and the Cochrane Library to identify full-text articles that reported the minimum dose delivered to 90% (D90) of the high-risk clinical target volume (HR-CTV) and the local tumor control rate for cervical cancer patients treated with adaptive brachytherapy. We searched the MeSH terms “Uterine Cervical Neoplasms” and all entry terms in the title or abstract to find articles about cervical cancer, constituting article set 1. Then, we searched all articles with “adaptive” or “IGABT” in the titles or abstracts to obtain article set 2 and those with “brachytherapy” in the titles or abstracts to obtain article set 3. Finally, we took the intersection of these three sets of articles. The search results were restricted to the English language (**Supplementary Table 1**). The last search of this systematic review was performed on February 1, 2022. We contacted the corresponding authors when full-text articles were not available.

### The Inclusion Criteria Were as Follows

Populations: patients with cervical cancer.

Interventions: EBRT with or without concurrent chemotherapy followed by 4D-IGABT.

Outcomes: the sample size, the equivalent dose in 2 Gy per fraction (EQD2) for a HR-CTV D90 and the local control rate.

### The Exclusion Criteria Were as Follows

1. Review articles and meeting abstracts.

2. Literature that focused on techniques (applicators, magnetic resonance, treatment plans, etc.), other treatment modalities (EBRT, surgery, chemotherapy, etc.), physics, case reports, etc.

3. Articles that used combinations of other therapy modalities, such as hysterectomy.

4. According to the affiliation of the study and the period of patients treated, the most recent and comprehensive data were included when the data originated from overlapping study samples.

The literature screening, data extraction, and dispute resolution processes have been described in detail in previous studies (8, 9). Regression analysis between HR-CTV D90 and local control was performed using a probit model in XLSTAT 2016 (Addinsoft, Paris, France). Furthermore, the studies that reported intermediate-risk clinical target volume (IR-CTV) were screened out from the included studies. A probit model analysis between IR-CTV D90 and local control was also performed. The use of the probit model in the XLSTAT software has been described in previous studies (8, 9). The statistical significance was set at the *p* < 0.05 level.

## Results

### Description of the Included Studies

The search of PubMed, Web of Science Core Collection, and Cochrane Library resulted in 232, 172, and 11 records, respectively. After a systematic literature search, title and abstract screening, and full-text screening, 19 studies involving 3,616 patients with IGABT met the inclusion criteria and were included in this systematic review and meta-regression analysis (**Supplementary Figure 1**). The main characteristics of the included studies are presented in **Tables 1**, **2**.

**Table 1:** Main characteristics of the included studies: study and concurrent chemoradiotherapy.

Study (Ref.)	Country	DR	*N*	WP EBRT dose (Gy)	CCRT (%)	IC/IS BT	Image guidance modality	Applicator optimization	HR-CTV size at BT
Pötter, R 2011 (10)	Austria	HDR	156	45–50.4	73.0%	44%	MRI	Positive	>5 cm (66.0%)
Lindegaard, JC 2013 (11)	Denmark	PDR	140	46 ± 2	79.0%	Yes	MRI 97.9%, CT 2.1%	Negative	6 (2–11) cm
Nomden, CN 2013 (12)	The Netherlands	Mixed*	46	45 (39.6–50.4)	74.0%	Yes	MRI	Negative	BT 1: (57 ± 37) cc
Kharofa, J 2014 (13)	USA	HDR	18	45–50.4	94.4%	0.0%	MRI	Positive	BT 1: 27 (17-72) cc; BT 5: 24 (12–60) cc
Castelnau-Marchand, P 2015 (14)	France	PDR	225	45–50.4	94.5%	2.2%	MRI 89.3%, CT 10.7%	Negative	(32.6 ± 21.8) cc
Lakosi, F 2015 (15)	Belgium	PDR	85	45–50.4**	100.0%	11.7%	MRI	Positive	(38.1 ± 27.6) cc
Ribeiro, I 2016 (16)	Belgium	PDR	170	45–50.4	90.0%	16.0%	MRI 85.3%, x-ray 9.4%, CT 5.3%	Negative	(35.7 ± 21) cc
Mahantshetty, U 2017 (17)	India	HDR	94	45	100.0%	Yes	MRI	Positive	(46.9 ± 24.6) cc
Simha, V 2018 (18)	India	HDR	60	46	100.0%	Yea	BT1,3: MRI***, BT2,4: CT	Negative	(27.5 ± 11.37) cc
Horeweg, N 2019 (19)	The Netherlands	HDR	155	45–48.6	100.0%	55.5%	MRI 72.3%, CT 3.9%, CT+MRI 23.9%	Negative	(4.6 ± 1.6) cm
Wu, PY 2019 (20)	Hong Kong, China	HDR	42	45	90.5%	62.9%	BT1,3: MR; BT2,4: CT	Positive	34.7 (12.3–155.1) cc
Möller S 2020 (21)	Sweden		138	51.9 (45–68.4)	94.2%	62.3%	MRI/CT	Negative	(54.5 ± 25.4) cc
Sundset, M 2021 (22)	Norway	HDR	65	45/50	75%	0.0%	CT	Negative	(38.3 ± 16.3) cc
Mahantshetty, U 2021 (23)	India	HDR	41	50	97.4%	100.0%	MRI (CT for day 2)	Positive	41 ± 21 cc
Murakami, N 2021 (24)	Japan, Thailand, Republic of Korea	HDR	162 307	30.6 (20–50.4) 30.6 (26–54)	90.7% 90.6%	31.5% 45.0%	CT 93.8%; MRI 6.2%	NR	36.3 ± 19.1 cc 4.5 (0–10.3) cm
Pötter, R 2021 (25)	Multicenter****		1416	45–50	94.3%	43.0%	MRI	Positive	28 (20–40) cc
Tharavichitkul, E 2021 (26)	Thailand	HDR	92	45–50.4	92.4%	15.2%	CT	Positive	5.3 (IQR:1.8) cm
le Guyader, M 2022 (27)	France	HDR	29/49/91	46 (43–50)	100.0%	100.0%	CT/CT + MRI	Positive	38 (29–40) cc 45 (29–82) cc 31 (13–69) cc
Vojtíšek R 2022 (28)	Czech Republic	HDR	131	45	79.4%	5.3%	CT and MRI	Negative	≥30 cc: 56.6%

*HDR (10.9%), PDR (84.8%), PDR+HDR (4.3%).

**10 Gy/5 fraction boost to primary disease for 18 (21.2%) patients.

***A non-contrast CT was acquired to facilitate the reconstruction of the applicator.

**** EMBRACE-I study done at 24 centers in Europe, Asia, and North America.

Ref., reference; DR, dose rate; N, number of patients; WP, whole pelvis; EBRT, external beam radiotherapy; CCRT, concurrent chemoradiotherapy; HDR, high dose rate; MRI, magnetic resonance imaging; PDR, pulse dose rate; CT, computed tomography; USA, United States of America; l TAUS, transabdominal ultrasound.

**Table 2:** Main characteristics of the included studies: dose volume parameters and tumor control.

Study (Ref.)	HR-CTV D90 (Gy_EQD2,10_)	IR-CTV D90 (Gy_EQD2,10_)	LCR (time)	D2cc to OARs (Gy_EQD2,3_)	Toxicity (time)
Pötter, R 2011 (10)	93 ± 13	NR	95.0% (3 y)	Bladder: 86 ± 17; rectum: 65 ± 9; sigmoid: 64 ± 9	Bladder G3+: 2%; rectum G3+: 4%; bowel G3+: 0%; vaginal G3+: 1% (3 y)
Lindegaard, JC 2013 (11)	91 (69–102)	68 (60-78)	91.0% (3 y)	Bladder: 71 (52–89); rectum: 64 (51–77); sigmoid: 65.5 (49–78)	GU G3+: 1%; GI G3+: 3%; vaginal G3+: 4% (3 y)
Nomden, CN 2013 (12)	84 ± 9	65 ± 5	93.0% (3 y)	Bladder: 83 ± 7; rectum: 66 ± 6; sigmoid: 61 ± 6; bowel: 64 ± 9	GU G3+: 2.2; GI G3+: 8.7%; vaginal G3+: 6.5% (41 m)
Kharofa, J 2014 (13)	88 (77–106)	NR	100.0% (2 y)	Bladder: 81 (72–90); rectum: 61 (54–69); sigmoid: 69 (61–75)	No G3+ toxicities (20 m)
Castelnau-Marchand, P 2015 (14)	80.4 ± 10.3	67.7 ± 6.1	86.4% (3 y)	Bladder: 71.1 ± 8.7; rectum: 62.1 ± 6.7; sigmoid: 60.0 ± 5.7	GU G3+: 4%; GI G3+: 4%; vaginal G3+: 2.7% (39 m)
Lakosi, F 2015 (15)	84.4 ± 9	69.1 ± 4.3	94.0% (3 y)	Bladder: 77.3 ± 10.5; rectum: 65 ± 6.8; sigmoid: 63 ± 7.9; bowel: 64 ± 9.1	GU G3+: 5%; GI G3+: 8%; vaginal G3+: 8% (3 y)
Ribeiro, I 2016 (16)	84.8 ± 8.36	68.7 ± 5.5	96.0% (37 m)	Bladder: 86.1 ± 8.6; rectum: 61.7 ± 7.8; sigmoid: 62.5 ± 9.2	GU G3+: 6%; rectal G3+: 5%; sigmoid G3+: 2%; vaginal G3+: 5% (37 m)
Mahantshetty, U 2017 (17)	88.3 ± 4.4	NR	90.1% (39 m)	Bladder: 85.7 ± 9.8; rectum: 65.5 ± 7.2; sigmoid: 67 ± 8.8	GU G3+: 11.6%; GI G3+: 8.7%; vaginal G3+: 4.3% (39 m)
Simha, V 2018 (18)	98.4 ± 9.6	76.4 ± 2.7	100.0% (50 m)	Bladder: 90.6 (mean); rectum: 70.2 (mean); sigmoid: 74.2 (mean)	GU G3+: 1.7%; GI G3+: 10.0% (50 m)
Horeweg, N 2019 (19)	83.8 (80.3–86.6)	65.0 (62.8–67.1)	90.4% (5 y)	Bladder: 78.6 (73–82.1); rectum: 67.7 (61.2–71.6); sigmoid: 62.5 (56.2–67); bowel: 55.1 (49.9–62.1)	GU G3+: 0.8%; rectal G3+: 3.3%; bowel G3+: 3.6%; vaginal G3+: 1.4% (5 y)
Wu, PY 2019 (20)	88.5 (63.4-113.4)	NR	90.0% (2 y)	Bladder: 83.1 (60.4–127.9); rectum: 67.5 (55.8–77.7); sigmoid: 69.0 (48.1–78.6); bowel: 68.9 (45.9–85.5)	No severe late toxicity (20 m)
Möller S 2020 (21)	88.4 ± 9.4	NR	97.1% (44 m)	BRS: 73.9 ± 7.4, 65.5 ± 7.2, 67.4 ± 7.7	GU G3+: 2.2%; bowel G3+: 0.7%; vaginal G3+: 0.0% (44 m)
Sundset, M 2021 (22)	80.2 ± 7.3	NR	90.8% (7.2 y)	NR*	GU G3+: 4; UI G3+: 5 (7.2 y)
Mahantshetty, U 2021 (23)	87.2 ± 3.6	NR	90.1% (2 y)	Bladder: 84.6 ± 6.9; rectum: 68.3 ± 5.7; sigmoid: 69.5 ± 5.9	Rectum G3+: 4.9% (22 m)
Murakami, N 2021 (24)	66.1(51.0–102.0) 67.5(41.3–97.3)	NR	94.4% (4 y) 85.0% (4 y)	64.8 (40.7–99.5) 51.1 (35.1–88.1) 67.6 (36.5–113.4) 57.3 (33.6–91.5)	GU G1+: 18.5%; GI G1+: 22.8%; vaginal G1+: 8.6% (4 y) GU G1+: 18.5%; GI G1+: 22.8%; vaginal G1+: 8.6% (4 y)
Pötter, R 2021 (25)	90 (85-94)	NR	92.0% (5 y)	Bladder: 76 (69–83); rectum: 62 (57–68); sigmoid: 64 (59–69); bowel: 58 (49–67)	GU G3+: 6.8%; GI G3+: 8.5%; vaginal G3+: 3.2% (5 y)
Tharavichitkul, E 2021 (26)	87.2 ± 3.2	NR	90.0% (2 y)	BRS: 84.0 ± 8.2; 68.8 ± 6.8; 69.8 ± 6.0	GI G3+: 2.2%; vaginal G3+: 2.2% (32 m)
le Guyader, M 2022 (27)	84 (82–90) 82 (72–89) 90 (77–98)	NR	75.9% (5 y) 83.7% (5 y) 91.2% (5 y)	BRS: 71 (66–81); 61 (55–69); 59 (54–67) BRS: 73 (61–79); 62 (54–78); 60 (49–76) BRS: 76 (58–85); 61 (47–79); 66 (50–79)	GU G3+: 10%; vaginal G3+: 21% (5 y) GU G3+: 6%; GI G3+: 10%; vaginal G3+: 4% (5 y) GU G3+: 7%; GI G3+: 5%; vaginal G3+: 10% (5 y)
Vojtíšek, R 2022 (28)	86.8 ± 5.5	NR	88.3% (3 y)	BRS: 75.3 ± 8.1; 60.2 ± 6.9; 63.9 ± 7.7	GU G3+: 6.9%; GI G3+: 5.3% (43 m)

*Shown as diagram, could not be accurately converted to numerical value.

Ref., reference; HR-CTV, high-risk clinical target volume; BT, brachytherapy; D90, the minimum dose delivered to 90% (of the target volume); LCR, local control rate; D2cc, the minimum dose delivered to the 2-cm^3^ volumes of the OARs that received the maximum dose; OARs, organs at risk; NR, not reported; y, years; m, months; GU, genitourinary; GI, gastrointestinal; BRS, bladder rectum sigmoid; IQR, interquartile range.

### Probit Model Analysis

**Figure 1:**
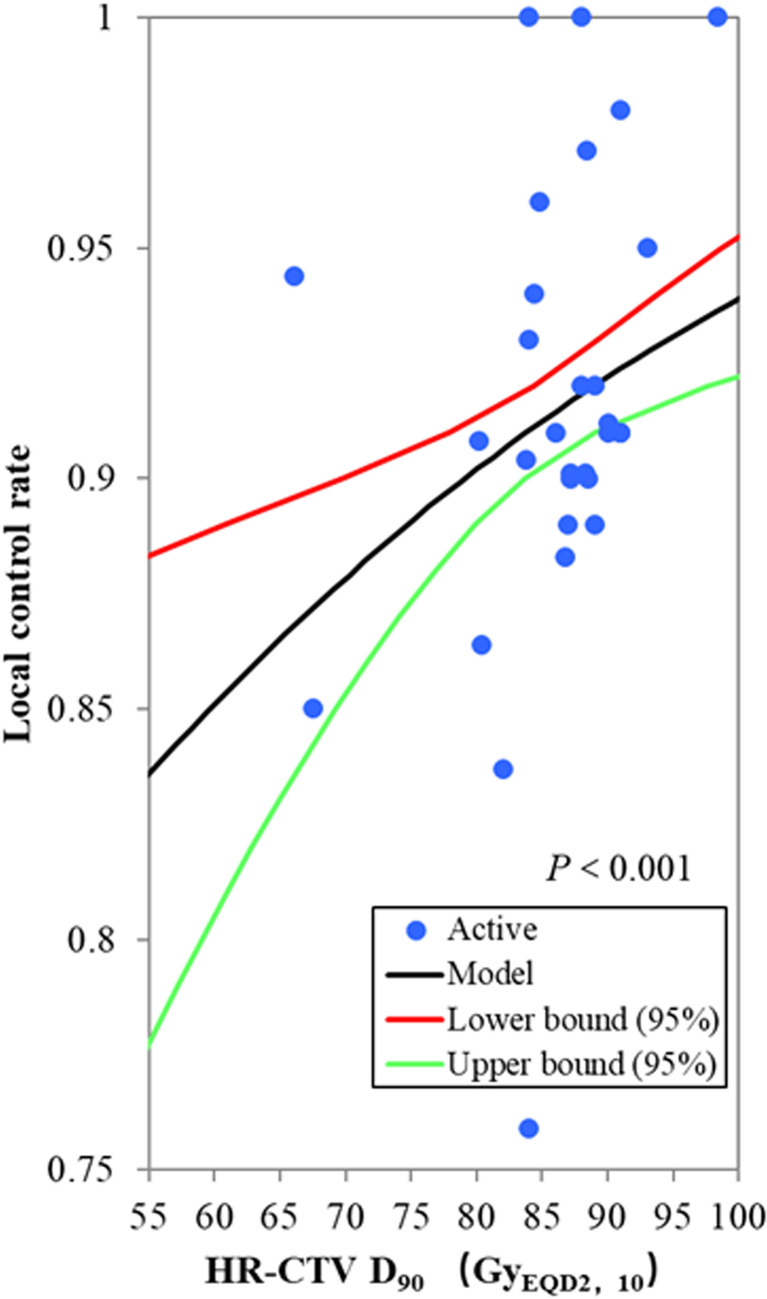
The probit model for the relationship between the HR-CTV D90 and local control probability. The blue active point represents the values of the HR-CTV D90 and the local control probability for each study. The HR-CTV D90 corresponding to a probability of 90% local control was 79.1 Gy_EQD2,10_ (95% confidence interval: 69.8–83.7 Gy_EQD2,10_), *p* < 0.001.

Among the 19 included studies, the sample sizes ranged from 18 to 1,416, with a total of 3,616 patients. Some studies reported HR-CTV D90 and local control rate for subgroups; therefore, the subgroup data were input into the probit model. The mean or median HR-CTV D90 for subgroups reported ranged from 66.1 to 98.4 Gy_EQD2,10_, and actuarial or crude local control rates from 75.9% to 100.0% were reported. The probit model showed a significant relationship between the HR-CTV D90 and the local control probability, *p* < 0.001. According to this model, the HR-CTV D90 corresponding to probabilities of 90% local control was 79.1 Gy_EQD2,10_ [95% confidence interval (CI): 69.8–83.7 Gy_EQD2,10_] (**Figure 1**). The limits for the prescribed dose of 85 Gy_EQD2,10_ theoretically warranted a 92.1% (95% CI: 90.2%–95.3%) local control rate. The planning aim dose of 90 Gy_EQD2,10_ for HR-CTV D90 recommended in the EMBRACE II study (29) corresponded to a 93.8% (95% CI: 91.2%–97.0%) local control probability.

**Figure 2:**
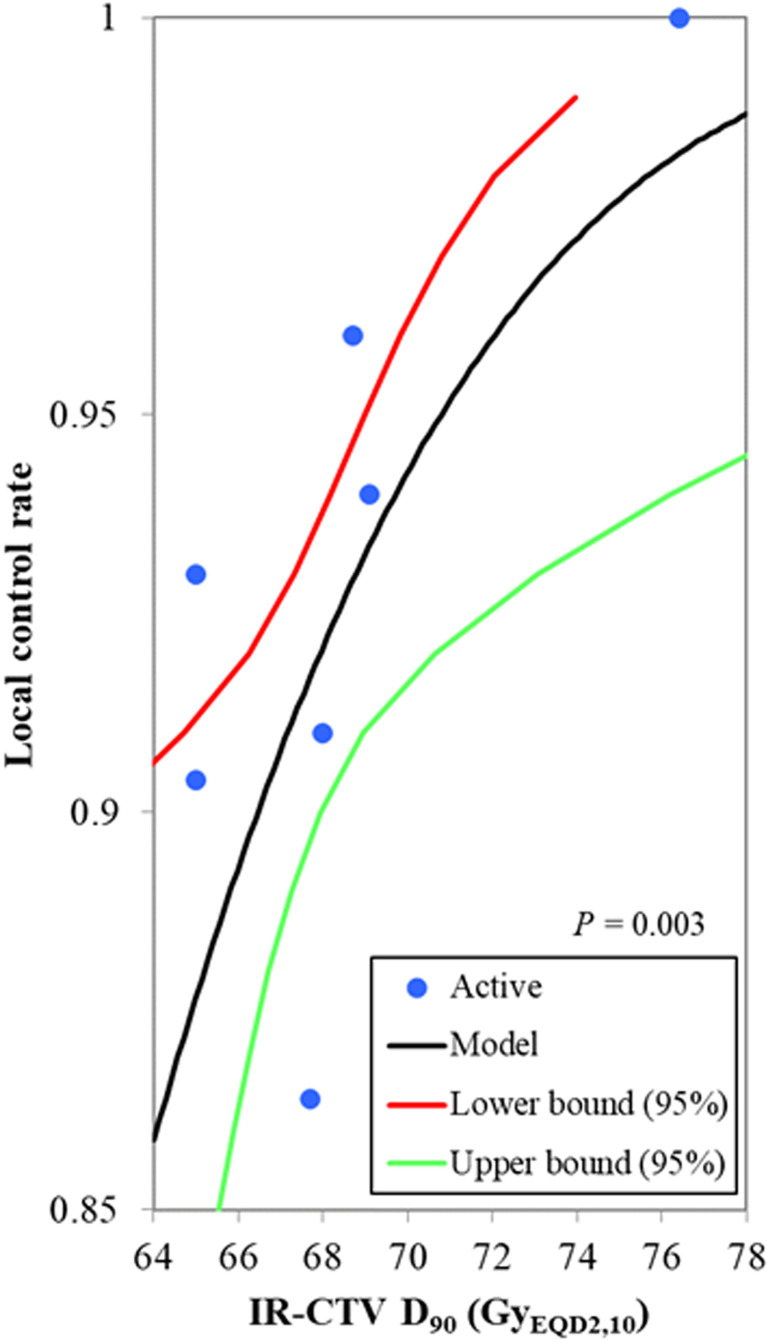
The probit model for the relationship between the IR-CTV D90 and the local control probability. The blue active point represents the values of the HR-CTV D90 and the local control probability for each study. The IR-CTV D90 corresponding to a probability of 90% local control was 66.5 Gy_EQD2,10_ (95% confidence interval: 62.8–67.9 Gy_EQD2,10_), *p* = 0.003.

Of the 19 studies included in our analysis, seven studies, including 881 patients, reported the mean or median value of the IR-CTV D90, ranging from 65.0 to 76.4 Gy_EQD2,10_. The local control rate was reported to range from 86.4% to 100.0%. The probit model showed a significant relationship between the IR-CTV D90 and the local control probability, *p* = 0.003. The IR-CTV D90 corresponding to probabilities of 90% and 95% local control was 66.5 Gy_EQD2,10_ (95% CI: 62.8–67.9 Gy_EQD2,10_) and 70.8 Gy_EQD2,10_ (95% CI: 69.0–79.9 Gy_EQD2,10_), respectively (**Figure 2**). The planning aim dose of 65 Gy_EQD2,10_ for IR-CTV D90 corresponded to an 87.2% (95% CI: 82.4%–91.8%) local control probability.

**Figure 3:**
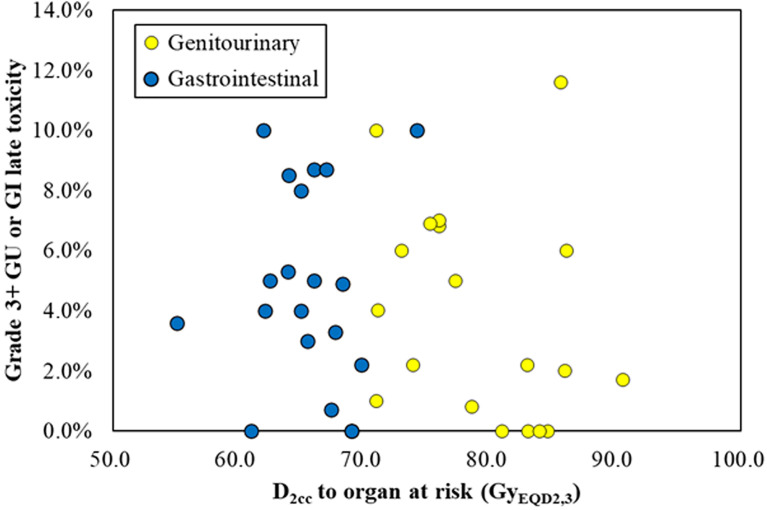
The scatter plot of late gastrointestinal (blue) and genitourinary (yellow) toxicity versus maximum D2cc of rectum, sigmoid, and small bowel and D2cc of bladder.

Of the 19 studies included in our analysis, the probability of grade 3 and above late gastrointestinal, genitourinary, and vaginal toxicity was reported from 0.0% to 10.0%, 0.0% to 11.6%, and 0.0% to 21.0%, respectively. The probit model showed no significant relationship between the bladder D2cc and the probability of grade 3 and above gastrointestinal toxicity, *p* = 0.245. There was also no significant relationship between the maximum D2cc of rectum, sigmoid, and small bowel and the probability of grade 3 and above gastrointestinal toxicity, *p* = 0.767. The scatter plot of late gastrointestinal and genitourinary toxicity versus maximum D2cc of rectum, sigmoid, and small bowel and D2cc of bladder is shown in **Figure 3**. We did not conduct probit model analysis between vaginal dose and vaginal toxicity due to the lack of sufficient data.

## Discussion

The concept of adaptive radiotherapy (ART) was first proposed by Yan Di et al. in 1997 (30). ART is a dynamic closed-loop feedback system with self-response and self-correction. It aims to provide more accurate targeting of disease by addressing the continuous changes in anatomy and/or physiology during treatment. The idea is to use imaging information to update the treatment plan daily rather than maintaining a static plan throughout the treatment course. In this way, ART offers the potential to improve delivery accuracy and adapt to anatomical and/or functional changes. The concept of ART was applied to image-guided brachytherapy to form IGABT (31).

### Why Do We Perform 4D Brachytherapy?

The implementation of adaptive brachytherapy is a more accurate treatment strategy with clinical requirements due to the very high doses per fraction, tumor regression, internal organ motion, and changes in organ filling.

In high-dose-rate (HDR) brachytherapy, the HR-CTV D90 per fraction is usually as high as 5 to 7 Gy (13, 20). Furthermore, the mean dose to the gross target volume (GTV) closer to the applicator is more than 100 Gy in total in combined EBRT and brachytherapy (32). For pulse-dose-rate (PDR) brachytherapy, its prescription dose per fraction is usually higher than that of HDR, approximately 10–15 Gy per fraction (11, 14). With such a high physical dose, the EQD2 is higher; thus, tumor regression is quite obvious even after only one fraction of brachytherapy. In our included studies, Kharofa et al. reported significant target volume reduction during brachytherapy (13). The median HR-CTV volume was 27 cc (range, 17–72 cc) at the first brachytherapy fraction and 24 cc (range, 12–60 cc) at the fifth brachytherapy insertion. The median volume reduction in HR-CTV from the first to last fraction of brachytherapy was 17% (range, 0%–41%). Target volume reduction is one of the important factors for adaptive strategy in brachytherapy. Similar target volume reduction has also been reported by other researchers. Christian et al. reported significant tumor regression in HDR brachytherapy (33). The mean volume of HR-CTV started at 43 cc in the first fraction and decreased to 29 cc in the last fraction, while the GTV decreased from 17 cc to 9 cc.

In addition to the changes in tumor size, there are also significant differences in the volume of OARs between fractions. Hellebust et al. evaluated the coefficient of variance (CV) of the bladder and rectum volume by using 3–6 CT scans in 14 patients who received HDR brachytherapy (34). The mean CVs of the bladder and rectum were 44.1% and 23.3%, respectively. Therefore, the mean CVs of doses for the bladder and rectum were also high, 17.5% and 15.0%, respectively.

In general, not only the volumes of the targets and OARs, but also the topography change. Meerschaert et al. evaluated the similarity between fraction 1 and fractions 2–5 for HR-CTV and OARs in 22 cervical cancer patients who received 5 fractions of HDR brachytherapy (35). The results showed that the mean Dice similarity coefficient values of the HR-CTV, bladder, rectum, and sigmoid were 0.4–0.5, 0.6–0.7, 0.5, and 0.3–0.4, respectively.

### How Do We Perform 4D Brachytherapy?

Based on the low similarity of the target and OARs, Meerschaert et al. objectively evaluated the difference between a single plan and an adaptive daily plan by defining a sparing factor, the ratio of the D2cc to the HR-CTV D90. Compared to the single plan, the sparing factors were lower for all OARs in the adaptive daily plan. This revealed the importance of replanning, which is one of the most common interventions for IGABT. Kirisits et al. compared individual treatment planning for each fraction and the use of only one treatment plan (36). The “single planning procedure” was simulated by matching the dose distribution of the first plan to the MRI dataset of each subsequent implantation. The use of only one treatment plan for several applications results in a higher dose to the target and OAR structures. Based on an analysis of 14 patients, the use of only one treatment plan for several applications results in a higher dose to the target and OAR structures. For some special cases, a lateral shift of the rectum and pronounced rotation of the ring applicator or a sigmoid nearer to the applicator due to tumor regression, a single plan will lead to severe dose underestimation.

The choice of applicator type depends on the individual anatomy and tumor spread at the time of brachytherapy. If the applicator in the previous brachytherapy session is not optimal, a different applicator has to be used in the subsequent fraction(s). Cheng et al. reported 119 cervical cancer patients with IGABT, and 38 patients (31.9%) changed the applicator during the course of HDR brachytherapy (37).

In the case of large tumors or unfavorable topography between the HR-CTV and OARs, the HR-CTV dose coverage may be compromised when using intracavitary (IC) brachytherapy (38). Skliarenko et al. analyzed the data of 20 patients with cervical cancer who received 4 fractions of HDR within two insertions using the tandem/ring with and without an interstitial applicator (39). The treatment plan geometry was adapted at the second insertion in 7 patients with suboptimal HR-CTV D90 and in three others with unsatisfactory OAR sparing. Interstitial needles were added in eight cases with IC brachytherapy, and additional needles were added in two cases with IC/IS brachytherapy. The HR-CTV D90 of the second insertion was significantly increased in 10 patients with the aid of interstitial needles. Seven of ten patients achieved the planning aim dose of 85 Gy due to adaptive intervention, and three others achieved a suboptimal dose of 80 Gy. The analysis from retroEMBRACE showed that combined intracavitary and interstitial (IC/IS) brachytherapy improved the local control rate by 10% at 3 years and 7% at 5 years for larger tumors (≥30 cc) (40). In the ambitious EMBRACE II study, increasing the use of IC/IS to improve the high-dose coverage of HR-CTV was one of the seven important interventions for patients with large tumors (29).

Several studies have shown that bladder fullness significantly affects the dose to the small bowel and bladder (41–44). Mahantshetty et al. compared the doses to OARs under different degrees of bladder filling (42). The results demonstrated that compared with an empty bladder, a significant decrease in the small bowel dose in full bladder filling was noted, and the bladder dose was higher. Yamashita et al. also quantified the effect of bladder volume on the dose distribution of IC brachytherapy for cervical cancer and a similar conclusion was obtained (41).

### Benefits of 4D Brachytherapy

IGABT takes into account various uncertain factors in the course of brachytherapy, such as tumor regression, internal organ motion, and bladder filling status, and achieves a more accurate treatment modality, so patients can benefit from 4D brachytherapy. Analysis from the retroEMBRACE study indeed showed that IGABT with IC/IS increased the therapeutic ratio of locally advanced cervical cancer (40).

Compared to the historical Vienna series, there is a relative reduction in pelvic recurrence of 65%–70% and a reduction in major morbidity, and the local control improvement seems to have an impact on cancer-specific survival and overall survival (10). An analysis of two studies from the University of Aarhus Denmark showed that compared with radiography-based brachytherapy, IGABT improved the 3-year overall survival rate from 63% to 79%, *p* = 0.005 (11). The EMBRACE-I study was internationally recognized as the most ambitious study worldwide to evaluate and benchmark image-guided adaptive brachytherapy. It resulted in effective and stable long-term local control across all stages of locally advanced cervical cancer, with a limited severe morbidity per organ (25).

### Dose-Effect Response

**Figure 4:**
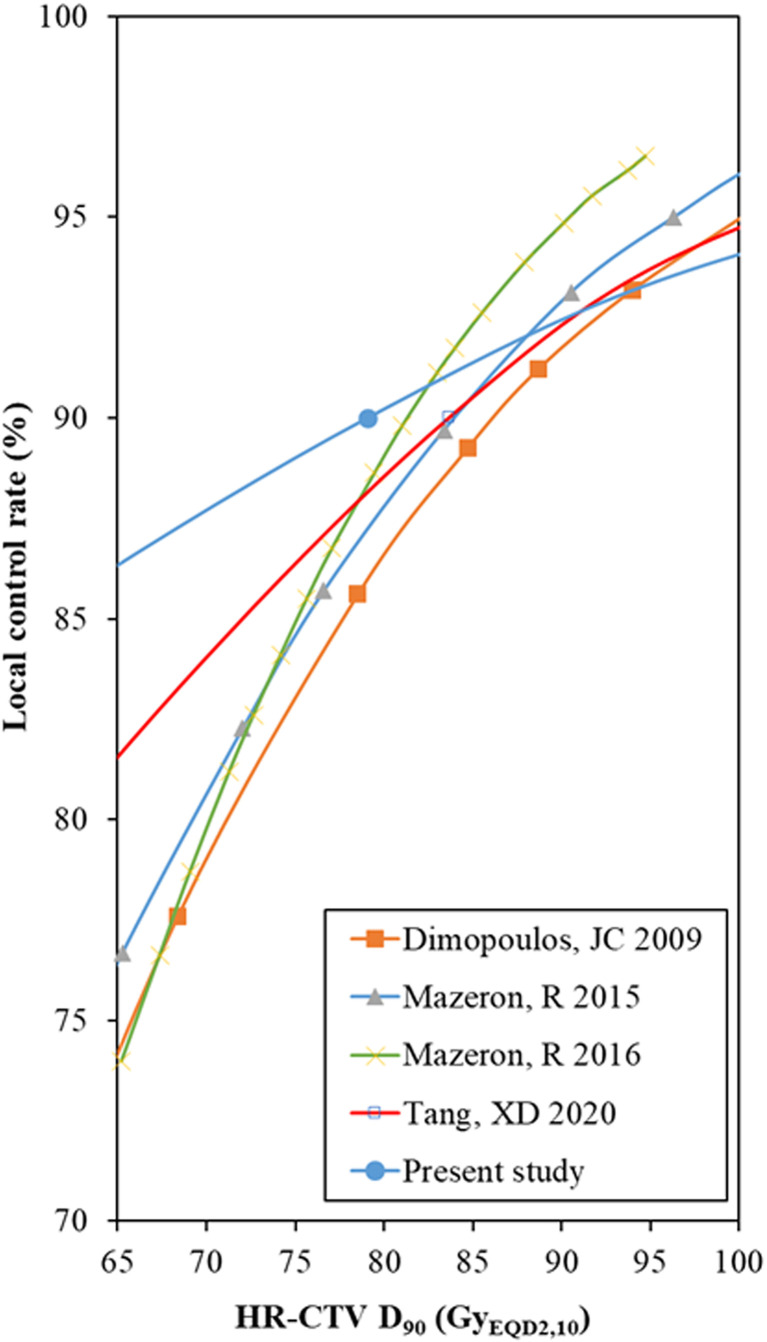
Comparison of the probit model results between the HR-CTV D90 and local control rate. HR-CTV = high-risk clinical target volume, D90 = the minimum dose delivered to 90% (of the target volume). *The data points of the probit models were manually extracted from the published figures.

To our knowledge, before our probit model analysis in the present study, there were four other studies that used regression analysis to estimate the local control rate: one for HDR brachytherapy, based on the data of 156 patients (45), one for PDR with 225 patients (46), one for a meta-regression of thirteen studies enrolling 1,299 patients (47), and one for a meta-regression of thirty-three studies including 2,893 patients (8) (**Figure 4**).

These estimates were based on independent individual patients or meta-regression analysis. However, individual patients and patients enrolled in meta-analysis studies had high heterogeneity, which was analyzed in our previous studies (8). At the same time, high-dose gradients in brachytherapy should be given more attention. Therefore, these conclusions should refer to each other, and take into account the current situation of the patient to determine the planning aim dose.

In our study, for the dose range from 65 to 80 Gy_EQD2,3_, the local control rate predicted by the probit model was higher than those in other previous studies. This is because there are some low-risk patient cohorts in the 19 studies included in this study. In particular, Murakami et al. (24) reported a multicenter study aimed at identifying a group of patients who can safely be treated by de-escalated treatment intensity. For the low-risk patient group (squamous cell carcinoma, tumor reduction ratio ≥29%, tumor size before brachytherapy ≤4 cm assessed by MRI, and total treatment time <9 weeks), a 4-year 94.4% local tumor control was achieved only by giving the mean dose to HR-CTV D90 66.1 (51.0–102.0) Gy. This result provided us with a reference, that is, strictly selected low-risk patients will achieve favorable local control with CTVHR D90 <80 Gy. In general, a higher local control rate was expected for a higher HR-CTV D90, especially for patients with larger tumor volumes or unfavorable stages (29, 48).

In the probit model analysis of this study, the 95% CIs of the HR-CTV D90 and IR-CTV D90 for predicting the 90% local control rate were small. This can be explained by the fact that the dose in 4D brachytherapy is more accurate and reliable. This benefits from the optimization of the applicator, the adjustment of the implant needles and bladder filling, and the balance of the cumulative dose between the applications.

The probit model showed no significant relationship between the severe late toxicity of OARs and the doses to OARs. This result is unexpected, but the conclusion does follow the data presented. The reasons for this result may include the following aspects. Firstly, when evaluating the total equivalent doses from external beam radiotherapy and from brachytherapy, we should pay more attention to the uncertainty caused by simplifications and assumptions. They include the following: (a) it is assumed that the organ walls adjacent to the applicator receiving a high brachytherapy absorbed dose (such as D0.1cc and D2cc) are irradiated with the full absorbed dose of external beam radiotherapy; (b) when adding doses for absolute OAR volumes (e.g., D2cc), it is assumed that the location of the given high-absorbed-dose volumes is identical for each brachytherapy fraction. Such assumptions are not necessarily valid for specific conditions. For assumption (a) techniques such as intensity-modulated radiation therapy or volumetric-modulated arc therapy that can produce more-pronounced absorbed-dose gradients in those regions of OARs irradiated to high absorbed dose during brachytherapy, such that the doses in these regions are extremely nonhomogeneous. The midline block or parametrial boost nearby will also bring a high-dose gradient, which makes it difficult to evaluate the D2cc of OARs from external beam radiotherapy. Assumption (b) overestimates the D2cc results to some extent. The degree of overestimation varies from patient to patient under different circumstances, which depends on the significance of organ movement and the difference of the change of applicator and interstitial needle between fractions. For organ movement, the peristalsis of the small bowel is relatively large, and it is affected by the bladder volume. For different bladder volumes, it varies greatly in D2cc of the small bowel, even in the area receiving D2cc. This is the reason why the doses of the bladder and the small bowel can be balanced by adjusting the volume of the bladder. At present, there are some methods of dose accumulation based on deformable image registration (49, 50). In theory, these methods can obtain better cumulative dose results, but they are also limited by the accuracy of deformable image registration. Unfortunately, for the studies included in this review, deformable image registration was not used. Secondly, in the probit model of OARs, the maximum D2cc of rectum, sigmoid, and small bowel is selected as the model input data, which has the problem of the accuracy of model input data. However, this is a compromise approach, because in many studies, gastrointestinal side effects from the rectum, colon, or small intestine are not reported in detail. In fact, some side effects, such as diarrhea, are difficult to distinguish from which organ, especially without colorectal endoscopic support. Finally, the heterogeneity of included patients limits the accuracy of the probit model to a certain extent. This has been mentioned in the limitations of the manuscript and a previous study. Although there is a lack of significant relationship, it is still necessary to strictly follow the dose constraints to OARs in IGABT. A new and more reliable method of dose accumulation for absolute OAR volumes needs to be broken through to obtain less uncertainty, so as to establish a more significant relationship between OAR dose and OAR toxicity.

### Limitations

The main limitation of this paper is the heterogeneity of the data, which has been described in detail in previous studies (8, 9). The local control rate of tumor is not only related to the dose to targets, but also directly related to the HR-CTV, the initial stage of the disease, the overall treatment time, and tumor width at diagnosis. Due to the few included literatures in this study, the local control rate was not reported according to the initial stage of the disease and the volume of HR-CTV; thus, the subgroup analysis could not be carried out. In addition, the incompleteness of paper retrieval is a limitation of this paper, as the keywords of the papers cannot be quickly retrieved in full text. Some studies using adaptive brachytherapy strategies were not included in our literature screening because the concepts “adaptive” and “IGABT” were not mentioned in the title or abstract.

## Conclusion

In conclusion, 4D brachytherapy takes into account uncertain factors such as tumor regression, internal organ motion, and organ filling, and it provides more accurate and more therapeutic ratio delivery through adaptive delineation and replanning, replacement of applicators, and the addition of interstitial needles. The dose volume effect relationship of 4D brachytherapy between the HR-CTV D90 and the local control rate provides an objective planning aim dose. A new and more reliable method of dose accumulation for absolute OAR volumes needs to be discovered to obtain less uncertainty.

## Data Availability Statement

The original contributions presented in the study are included in the article/**Supplementary Material**. Further inquiries can be directed to the corresponding author.

## Author Contributions

HZ and FL conceived the study. HZ, DS, MB, and SL conducted research, extracted data, performed statistical analysis, and wrote this paper. FL, DS, and HZ revised and corrected the manuscript. All authors contributed to the article and approved the submitted version.

## Conflict of Interest

Author MB was employed by Guowen Medical Corporation Changchun Hospital.

The remaining authors declare that the research was conducted in the absence of any commercial or financial relationships that could be construed as a potential conflict of interest.

## Publisher’s Note

All claims expressed in this article are solely those of the authors and do not necessarily represent those of their affiliated organizations, or those of the publisher, the editors and the reviewers. Any product that may be evaluated in this article, or claim that may be made by its manufacturer, is not guaranteed or endorsed by the publisher.
