# Supplementary figures and images for: Four-Dimensional Image-Guided Adaptive Brachytherapy for Cervical Cancer: A Systematic Review and Meta-Regression Analysis
Source: Front Oncol. 2022 Jul 4;12:870570. doi: 10.3389/fonc.2022.870570 (PMC9291247; doi:10.3389/fonc.2022.870570)

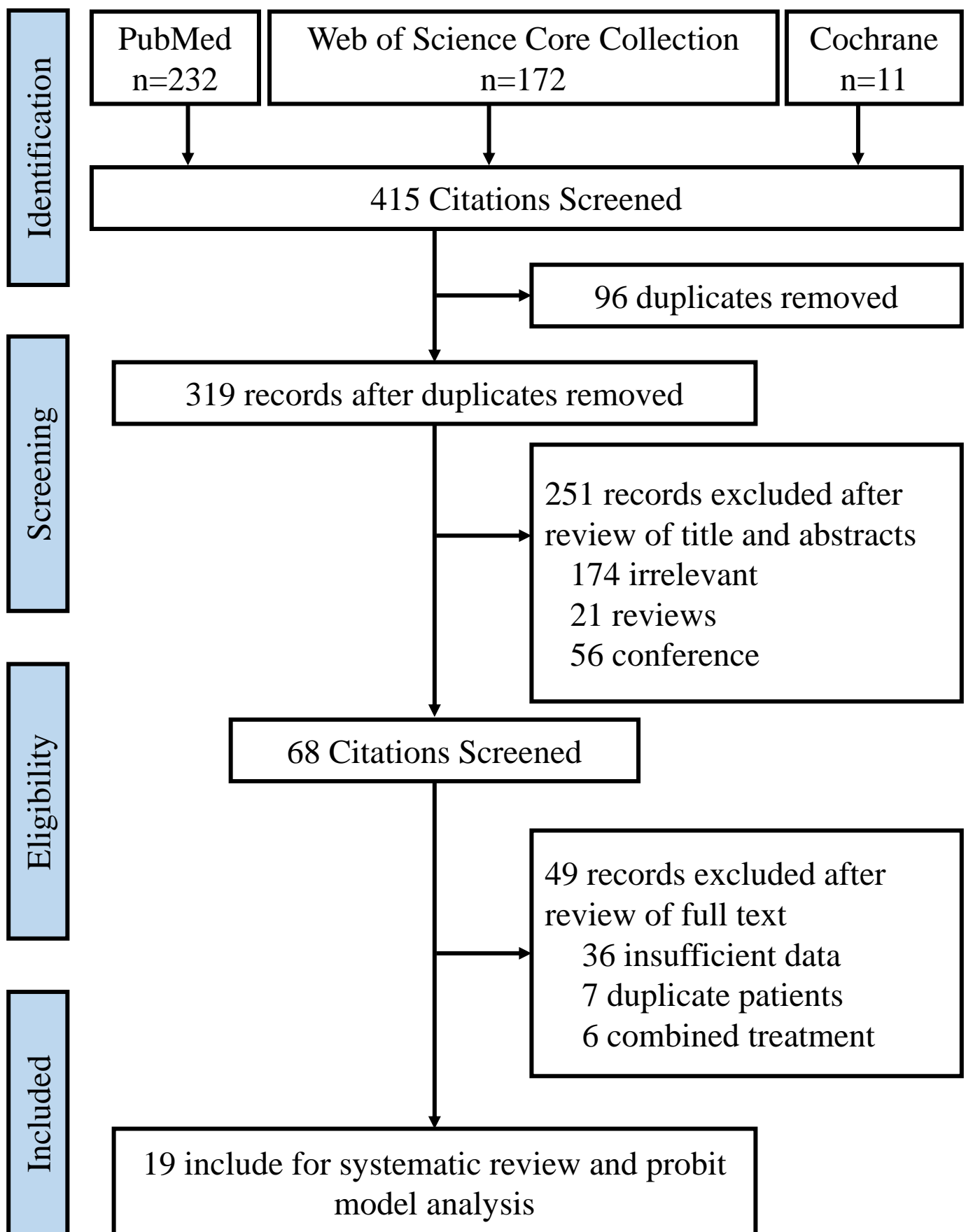

Figure S1. PRISMA Flow diagram of the included studies.

Supplement: Supplementary file 1 [file DataSheet_1.pdf]
