# Supplementary material for: Four-Dimensional Image-Guided Adaptive Brachytherapy for Cervical Cancer: A Systematic Review and Meta-Regression Analysis
Source: Front Oncol. 2022 Jul 4;12:870570. doi: 10.3389/fonc.2022.870570 (PMC9291247; doi:10.3389/fonc.2022.870570)
Supplement: Supplementary file 2 [file Table_1.docx]

| Supplemental Table 1. Search strategies of databases | | |
| --- | --- | --- |
| **Databases** | **Search strategies** | **Search outcomes** |
| **The PubMed Database** | #1 Search Uterine Cervical Neoplasms [MeSH Terms]  #2 Search ((((((((((((((((((((((((((Cervical Neoplasm, Uterine[Title/Abstract]) OR Cervical Neoplasms, Uterine[Title/Abstract]) OR Neoplasm, Uterine Cervical[Title/Abstract]) OR Neoplasms, Uterine Cervical[Title/Abstract]) OR Uterine Cervical Neoplasm[Title/Abstract]) OR Neoplasms, Cervical[Title/Abstract]) OR Cervical Neoplasms[Title/Abstract]) OR Cervical Neoplasm[Title/Abstract]) OR Neoplasm, Cervical[Title/Abstract]) OR Neoplasms, Cervix[Title/Abstract]) OR Cervix Neoplasms[Title/Abstract]) OR Cervix Neoplasm[Title/Abstract]) OR Neoplasm, Cervix[Title/Abstract]) OR Cancer of the Uterine Cervix[Title/Abstract]) OR Cancer of the Cervix[Title/Abstract]) OR Cervical Cancer[Title/Abstract]) OR Uterine Cervical Cancer[Title/Abstract]) OR Cancer, Uterine Cervical[Title/Abstract]) OR Cancers, Uterine Cervical[Title/Abstract]) OR Cervical Cancer, Uterine[Title/Abstract]) OR Cervical Cancers, Uterine[Title/Abstract]) OR Uterine Cervical Cancers[Title/Abstract]) OR Cancer of Cervix[Title/Abstract]) OR Cervix Cancer[Title/Abstract]) OR Cancer, Cervix[Title/Abstract]) OR Cancers, Cervix[Title/Abstract]) OR Carcinoma of the Cervix[Title/Abstract]  #3 Search (#1) OR #2  #4 Search (adaptive[Title/Abstract]) OR (IGABT[Title/Abstract])  #5 Search (brachytherapy [Title/Abstract])  #6 Search ((#3) AND (#4)) AND (#5)  Filter: Language: English | 80,295  81,061  105,147  180,746  19,321  238  232 |
| **Web of Science Core Collection** | #1 TOPIC: (Uterine Cervical Neoplasms)  #2 TI=(Cervical Neoplasm, Uterine) OR TI=(Cervical Neoplasms, Uterine) OR TI=(Neoplasm, Uterine Cervical) OR TI=(Neoplasms, Uterine Cervical) OR TI=(Uterine Cervical Neoplasm) OR TI=(Neoplasms, Cervical) OR TI=(Cervical Neoplasms) OR TI=(Cervical Neoplasm) OR TI=(Neoplasm, Cervical) OR TI=(Neoplasms, Cervix) OR TI=(Cervix Neoplasms) OR TI=(Cervix Neoplasm) OR TI=(Neoplasm, Cervix) OR TI=(Cancer of the Uterine Cervix) OR TI=(Cancer of the Cervix) OR TI=(Cervical Cancer) OR TI=(Uterine Cervical Cancer) OR TI=(Cancer, Uterine Cervical) OR TI=(Cancers, Uterine Cervical) OR TI=(Cervical Cancer, Uterine) OR TI=(Cervical Cancers, Uterine) OR TI=(Uterine Cervical Cancers) OR TI=(Cancer of Cervix) OR TI=(Cervix Cancer) OR TI=(Cancer, Cervix) OR TI=(Cancers, Cervix) OR TI=(Carcinoma of the Cervix)  #3 #1 OR #2  #4 TI=(adaptive) OR TI=(IGABT)  #5 TI=(brachytherapy)  #6 #3 AND #4 AND #5  Filter: Language: English | 45,513  34,960  60,726  194,703  17,982  174  172 |
| **The Cochrane Library** | #1 MeSH descriptor: [Uterine Cervical Neoplasms] explode all trees  #2 (Uterine Cervical Neoplasm): ti, ab, kw  #3 (Cervical Neoplasms): ti, ab, kw  #4 (Cervical Neoplasm): ti, ab, kw  #5 (Cervix Neoplasms): ti, ab, kw  #6 (Cervix Neoplasm): ti, ab, kw  #7 (Cancer of the Uterine Cervix): ti, ab, kw  #8 (Cancer of the Cervix): ti, ab, kw  #9 (Cervical Cancer): ti, ab, kw  #10 (Uterine Cervical Cancer): ti, ab, kw  #11 (Uterine Cervical Cancers): ti, ab, kw  #12 (Cervix Cancer): ti, ab, kw  #13 (Cancers, Cervix): ti, ab, kw  #14 (Carcinoma of the Cervix): ti, ab, kw  #15 #1 OR #2 OR #3 OR #4 OR #5 OR #6 OR #7 OR #8 OR #9 OR #10 OR #11 OR #12 OR #13 OR #14  #16 (adaptive): ti, ab, kw  #17 (IGABT): ti, ab, kw  #18 (brachytherapy): ti, ab, kw  #19 #15 AND #16 AND #18 | 2,234  551  2,916  787  1,183  496  2,674  3,152  6,073  3,703  388  3,271  375  1,687  7,459  9,620  2  2,407  11 |
